# 4-(3-Methoxy­phen­yl)-1-(2-oxoindolin-3-yl­idene)thio­semicarbazide

**DOI:** 10.1107/S1600536810018052

**Published:** 2010-05-22

**Authors:** Humayun Pervez, Mohammad S. Iqbal, Naveeda Saira, Muhammad Yaqub, M. Nawaz Tahir

**Affiliations:** a Department of Chemistry, Bahauddin Zakariya University, Multan 60800, Pakistan; b Department of Chemistry, Government College University, Lahore, Pakistan; c Department of Physics, University of Sargodha, Sargodha, Pakistan

## Abstract

In the title compound, C_16_H_14_N_4_O_2_S, intra­molecular N—H⋯N hydrogen bonding forms an *S*(5) ring, whereas N—H⋯O and C—H⋯S inter­actions complete *S*(6) ring motifs. In the crystal, mol­ecules form inversion dimers due to N—H⋯O inter­actions. The dimers are inter­linked through N—H⋯S hydrogen bonds and π–π inter­actions occur with a centroid–centroid distance of 3.8422 (11) Å between the meth­oxy-containing benzene ring and the five-membered heterocyclic ring.

## Related literature

For the preparation and structures of biologically important *N* ^4^-aryl-substituted isatin-3-thio­semicarbazones, see: Pervez *et al.* (2007 ▶, 2008 ▶, 2009 ▶, 2010*a* ▶). For a related structure, see: Pervez *et al.* (2010*b* ▶). For graph-set notation, see: Bernstein *et al.* (1995 ▶).
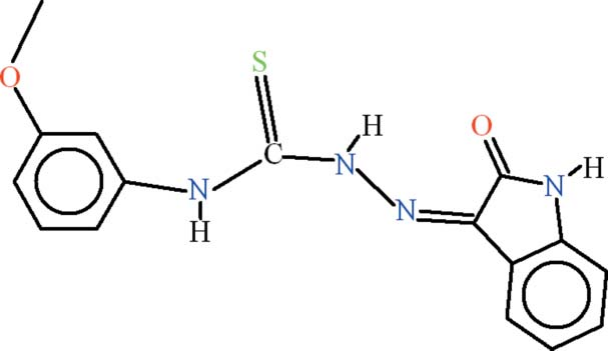


## Experimental

### 

#### Crystal data

C_16_H_14_N_4_O_2_S
*M* *_r_* = 326.37
Monoclinic, 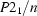

*a* = 15.1793 (5) Å
*b* = 7.2473 (2) Å
*c* = 15.4764 (5) Å
β = 111.179 (2)°
*V* = 1587.55 (9) Å^3^
*Z* = 4
Mo *K*α radiation
μ = 0.22 mm^−1^
*T* = 296 K
0.34 × 0.22 × 0.20 mm

#### Data collection

Bruker Kappa APEXII CCD diffractometer
Absorption correction: multi-scan (*SADABS*; Bruker, 2005 ▶) *T*_min_ = 0.946, *T*_max_ = 0.960
13861 measured reflections
3925 independent reflections
2898 reflections with *I* > 2σ(*I*)
*R*_int_ = 0.028

#### Refinement

*R*[*F* ^2^ > 2σ(*F* ^2^)] = 0.044
*wR*(*F* ^2^) = 0.125
*S* = 1.04
3925 reflections
209 parameters
H-atom parameters constrained
Δρ_max_ = 0.36 e Å^−3^
Δρ_min_ = −0.35 e Å^−3^

### 

Data collection: *APEX2* (Bruker, 2007 ▶); cell refinement: *SAINT* (Bruker, 2007 ▶); data reduction: *SAINT*; program(s) used to solve structure: *SHELXS97* (Sheldrick, 2008 ▶); program(s) used to refine structure: *SHELXL97* (Sheldrick, 2008 ▶); molecular graphics: *ORTEP-3 for Windows* (Farrugia, 1997 ▶) and *PLATON* (Spek, 2009 ▶); software used to prepare material for publication: *WinGX* (Farrugia, 1999 ▶) and *PLATON*.

## Supplementary Material

Crystal structure: contains datablocks global, I. DOI: 10.1107/S1600536810018052/si2262sup1.cif

Structure factors: contains datablocks I. DOI: 10.1107/S1600536810018052/si2262Isup2.hkl

Additional supplementary materials:  crystallographic information; 3D view; checkCIF report

## Figures and Tables

**Table 1:** Hydrogen-bond geometry (Å, °)

*D*—H⋯*A*	*D*—H	H⋯*A*	*D*⋯*A*	*D*—H⋯*A*
N1—H1⋯O1^i^	0.86	2.04	2.875 (2)	164
N3—H3*A*⋯O1	0.86	2.06	2.7441 (19)	136
N4—H4*A*⋯N2	0.86	2.19	2.620 (2)	110
N4—H4*A*⋯S1^ii^	0.86	2.87	3.5806 (16)	141
C11—H11⋯S1	0.93	2.74	3.212 (2)	112

Symmetry codes: (i) 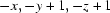; (ii) 
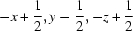
.

**Fig. 1.**
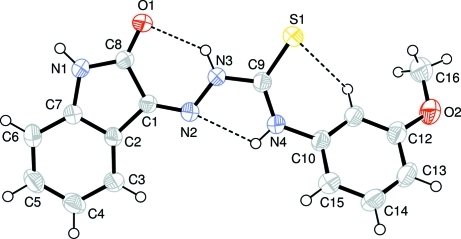
View of the title compound with the atom numbering scheme. The thermal ellipsoids are drawn at the 50% probability level. H-atoms are shown by small circles of arbitrary radii. The dotted lines indicate the intra-molecular H-bondings.

**Fig. 2.**
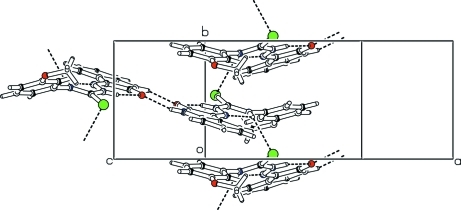
The partial packing (PLATON; Spek, 2009) which shows that molecules form dimers which are interlinked.

## supplementary crystallographic information

### Comment

In continuation of our work on the synthesis of medicinally important organic molecules (Pervez *et al.*, 2007, 2008, 2009, 2010*a*), we report herein the structure and synthesis of the title compound (I, Fig. 1).

The crystal structure of (II) i.e. 4-(2-fluorophenyl)-1-(2-oxoindolin-3-ylidene)thiosemicarbazide has been published (Pervez *et al.*, 2010*b*). The title compound (I) differs from (II) due to the attachment of methoxy group at position-3 instead of fluoro at position-2 of the phenyl ring substituted at N^4^ of the thiosemicarbazone moiety.

In (I) the 2-oxoindolin A (C1–C8/N1/O1), thiosemicarbazide B (N2/N3/C9/S1/N4) and the 3-methoxyphenyl C (C10—C16/O2) are planar with r. m. s. deviations of 0.0178, 0.0244 and 0.0149 Å, respectively. The dihedral angle between A/B, A/C and B/C is 8.71 (5)°, 33.59 (3)° and 39.32 (3)°, respectively. Due to intramolecular H-bondings (Table 1, Fig. 1), one S(5) and two S(6) (Bernstein *et al.*, 1995) ring motifs are formed. The molecules are dimerised (Fig. 2) due to intermolecular H-bonding of N—H···O type with R_2_^2^(8) ring motifs. The dimers are interlinked through N—H···S type of H-bonding. There exist π···π interaction at a distance of 3.8422 (11) Å between the benzene ring (C10—C15) and the heterocyclic ring (N1/C7/C2/C1/C8).

### Experimental

To a hot solution of isatin (0.74 g, 5.0 mmol) in ethanol (10 ml) containing a few drops of glacial acetic acid was added 4-(3-methoxyphenyl)thiosemicarbazide (0.99 g, 5.0 mmol) dissolved in ethanol (10 ml) under stirring. The reaction mixture was then heated under reflux for 2 h. The yellow crystalline solid formed during refluxing was collected by suction filtration. Thorough washing with hot ethanol followed by ether afforded the target compound (I) in pure form (1.25 g, 77%), m. p. 477 K (d). The single crystals of (I) were grown in acetone by slow evaporation at room temperature.

### Crystal data

**Table:**

C_16_H_14_N_4_O_2_S	*F*(000) = 680
*M**_r_* = 326.37	*D*_x_ = 1.366 Mg m^−^^3^
Monoclinic, *P*2_1_/*n*	Mo *K*α radiation, λ = 0.71073 Å
Hall symbol: -P 2yn	Cell parameters from 2898 reflections
*a* = 15.1793 (5) Å	θ = 3.2–28.3°
*b* = 7.2473 (2) Å	µ = 0.22 mm^−^^1^
*c* = 15.4764 (5) Å	*T* = 296 K
β = 111.179 (2)°	Prism, yellow
*V* = 1587.55 (9) Å^3^	0.34 × 0.22 × 0.20 mm
*Z* = 4	

### Data collection

**Table:**

Bruker Kappa APEXII CCD diffractometer	3925 independent reflections
Radiation source: fine-focus sealed tube	2898 reflections with *I* > 2σ(*I*)
graphite	*R*_int_ = 0.028
Detector resolution: 7.40 pixels mm^-1^	θ_max_ = 28.3°, θ_min_ = 3.2°
ω scans	*h* = −20→20
Absorption correction: multi-scan (*SADABS*; Bruker, 2005)	*k* = −9→9
*T*_min_ = 0.946, *T*_max_ = 0.960	*l* = −20→19
13861 measured reflections	

### Refinement

**Table:**

Refinement on *F*^2^	Primary atom site location: structure-invariant direct methods
Least-squares matrix: full	Secondary atom site location: difference Fourier map
*R*[*F*^2^ > 2σ(*F*^2^)] = 0.044	Hydrogen site location: inferred from neighbouring sites
*wR*(*F*^2^) = 0.125	H-atom parameters constrained
*S* = 1.04	*w* = 1/[σ^2^(*F*_o_^2^) + (0.0539*P*)^2^ + 0.6227*P*] where *P* = (*F*_o_^2^ + 2*F*_c_^2^)/3
3925 reflections	(Δ/σ)_max_ < 0.001
209 parameters	Δρ_max_ = 0.36 e Å^−^^3^
0 restraints	Δρ_min_ = −0.35 e Å^−^^3^

### Special details

**Table:**

Geometry. All esds (except the esd in the dihedral angle between two l.s. planes) are estimated using the full covariance matrix. The cell esds are taken into account individually in the estimation of esds in distances, angles and torsion angles; correlations between esds in cell parameters are only used when they are defined by crystal symmetry. An approximate (isotropic) treatment of cell esds is used for estimating esds involving l.s. planes.
Refinement. Refinement of *F*^2^ against ALL reflections. The weighted *R*-factor *wR* and goodness of fit *S* are based on *F*^2^, conventional *R*-factors *R* are based on *F*, with *F* set to zero for negative *F*^2^. The threshold expression of *F*^2^ > σ(*F*^2^) is used only for calculating *R*-factors(gt) *etc*. and is not relevant to the choice of reflections for refinement. *R*-factors based on *F*^2^ are statistically about twice as large as those based on *F*, and *R*- factors based on ALL data will be even larger.

### Fractional atomic coordinates and isotropic or equivalent isotropic displacement parameters (Å^2^)

**Table:**

	*x*	*y*	*z*	*U*_iso_*/*U*_eq_	
C1	0.19826 (11)	0.3610 (2)	0.45348 (11)	0.0329 (3)	
C2	0.26044 (11)	0.3126 (2)	0.54720 (11)	0.0345 (3)	
C3	0.35347 (12)	0.2547 (3)	0.58412 (12)	0.0434 (4)	
H3	0.3882	0.2332	0.5464	0.052*	
C4	0.39313 (14)	0.2298 (3)	0.67919 (13)	0.0515 (5)	
H4	0.4555	0.1908	0.7057	0.062*	
C5	0.34181 (15)	0.2617 (3)	0.73516 (13)	0.0529 (5)	
H5	0.3705	0.2446	0.7988	0.063*	
C6	0.24859 (14)	0.3187 (3)	0.69884 (12)	0.0491 (5)	
H6	0.2140	0.3403	0.7367	0.059*	
C7	0.20928 (11)	0.3419 (2)	0.60448 (11)	0.0382 (4)	
C8	0.10524 (11)	0.4125 (2)	0.46058 (11)	0.0359 (4)	
C9	0.17432 (11)	0.4498 (2)	0.22293 (11)	0.0360 (4)	
C10	0.30202 (12)	0.3962 (2)	0.16100 (12)	0.0388 (4)	
C11	0.24941 (12)	0.3445 (2)	0.07119 (12)	0.0407 (4)	
H11	0.1865	0.3103	0.0552	0.049*	
C12	0.29128 (13)	0.3440 (3)	0.00497 (13)	0.0446 (4)	
C13	0.38568 (14)	0.3931 (3)	0.02993 (15)	0.0539 (5)	
H13	0.4139	0.3948	−0.0143	0.065*	
C14	0.43705 (14)	0.4392 (3)	0.11998 (16)	0.0554 (5)	
H14	0.5006	0.4689	0.1366	0.066*	
C15	0.39605 (13)	0.4423 (3)	0.18678 (14)	0.0477 (4)	
H15	0.4313	0.4748	0.2476	0.057*	
C16	0.14733 (15)	0.2526 (3)	−0.11290 (15)	0.0613 (6)	
H16A	0.1142	0.3578	−0.1023	0.092*	
H16B	0.1387	0.1496	−0.0777	0.092*	
H16C	0.1230	0.2222	−0.1776	0.092*	
N1	0.11680 (10)	0.3984 (2)	0.55058 (10)	0.0429 (4)	
H1	0.0733	0.4210	0.5725	0.051*	
N2	0.22156 (9)	0.36975 (19)	0.38155 (9)	0.0351 (3)	
N3	0.15482 (9)	0.4293 (2)	0.30214 (9)	0.0374 (3)	
H3A	0.0992	0.4548	0.3013	0.045*	
N4	0.26118 (10)	0.3955 (2)	0.23067 (10)	0.0416 (4)	
H4A	0.2966	0.3552	0.2842	0.050*	
O1	0.03312 (8)	0.45962 (19)	0.39631 (8)	0.0452 (3)	
O2	0.24526 (10)	0.2944 (2)	−0.08484 (9)	0.0610 (4)	
S1	0.09025 (3)	0.53985 (8)	0.13109 (3)	0.04856 (16)	

### Atomic displacement parameters (Å^2^)

**Table:**

	*U*^11^	*U*^22^	*U*^33^	*U*^12^	*U*^13^	*U*^23^
C1	0.0311 (7)	0.0360 (8)	0.0318 (8)	0.0010 (6)	0.0115 (6)	−0.0041 (6)
C2	0.0357 (8)	0.0370 (9)	0.0304 (8)	0.0024 (6)	0.0115 (6)	−0.0028 (6)
C3	0.0409 (9)	0.0480 (10)	0.0403 (9)	0.0111 (7)	0.0136 (7)	−0.0015 (8)
C4	0.0456 (10)	0.0572 (12)	0.0423 (10)	0.0146 (9)	0.0045 (8)	0.0006 (9)
C5	0.0585 (12)	0.0603 (12)	0.0312 (9)	0.0100 (9)	0.0060 (8)	0.0039 (8)
C6	0.0547 (11)	0.0628 (12)	0.0320 (9)	0.0061 (9)	0.0182 (8)	−0.0004 (8)
C7	0.0370 (8)	0.0444 (9)	0.0329 (8)	0.0019 (7)	0.0123 (7)	−0.0013 (7)
C8	0.0306 (7)	0.0442 (9)	0.0335 (8)	−0.0012 (6)	0.0121 (6)	−0.0050 (7)
C9	0.0361 (8)	0.0396 (9)	0.0331 (8)	−0.0034 (7)	0.0136 (7)	−0.0010 (7)
C10	0.0408 (9)	0.0398 (9)	0.0411 (9)	0.0054 (7)	0.0211 (7)	0.0075 (7)
C11	0.0412 (9)	0.0431 (10)	0.0421 (9)	0.0028 (7)	0.0200 (7)	0.0032 (7)
C12	0.0518 (10)	0.0442 (10)	0.0445 (10)	0.0062 (8)	0.0253 (8)	0.0032 (8)
C13	0.0548 (11)	0.0636 (13)	0.0570 (12)	0.0033 (10)	0.0367 (10)	0.0059 (10)
C14	0.0414 (10)	0.0658 (13)	0.0656 (13)	−0.0035 (9)	0.0272 (10)	0.0046 (10)
C15	0.0415 (9)	0.0565 (12)	0.0465 (10)	0.0005 (8)	0.0174 (8)	0.0036 (9)
C16	0.0634 (13)	0.0705 (15)	0.0487 (12)	−0.0020 (11)	0.0187 (10)	−0.0053 (10)
N1	0.0337 (7)	0.0646 (10)	0.0332 (7)	0.0050 (7)	0.0154 (6)	−0.0018 (7)
N2	0.0345 (7)	0.0396 (8)	0.0317 (7)	0.0018 (6)	0.0124 (6)	−0.0014 (6)
N3	0.0303 (6)	0.0520 (9)	0.0304 (7)	0.0027 (6)	0.0114 (5)	0.0015 (6)
N4	0.0355 (7)	0.0579 (10)	0.0330 (7)	0.0072 (6)	0.0143 (6)	0.0079 (6)
O1	0.0303 (6)	0.0680 (9)	0.0342 (6)	0.0061 (5)	0.0081 (5)	−0.0031 (6)
O2	0.0614 (9)	0.0838 (11)	0.0445 (8)	0.0019 (8)	0.0272 (7)	−0.0089 (7)
S1	0.0377 (2)	0.0708 (4)	0.0363 (2)	0.0074 (2)	0.01228 (18)	0.0104 (2)

### Geometric parameters (Å, °)

**Table:**

C1—N2	1.286 (2)	C10—C11	1.381 (2)
C1—C2	1.459 (2)	C10—N4	1.425 (2)
C1—C8	1.503 (2)	C11—C12	1.387 (2)
C2—C3	1.383 (2)	C11—H11	0.9300
C2—C7	1.390 (2)	C12—O2	1.359 (2)
C3—C4	1.386 (3)	C12—C13	1.389 (3)
C3—H3	0.9300	C13—C14	1.370 (3)
C4—C5	1.378 (3)	C13—H13	0.9300
C4—H4	0.9300	C14—C15	1.386 (3)
C5—C6	1.384 (3)	C14—H14	0.9300
C5—H5	0.9300	C15—H15	0.9300
C6—C7	1.374 (2)	C16—O2	1.422 (3)
C6—H6	0.9300	C16—H16A	0.9600
C7—N1	1.410 (2)	C16—H16B	0.9600
C8—O1	1.2319 (19)	C16—H16C	0.9600
C8—N1	1.343 (2)	N1—H1	0.8600
C9—N4	1.339 (2)	N2—N3	1.3504 (18)
C9—N3	1.369 (2)	N3—H3A	0.8600
C9—S1	1.6624 (17)	N4—H4A	0.8600
C10—C15	1.377 (2)		
			
N2—C1—C2	126.25 (14)	C10—C11—H11	120.3
N2—C1—C8	127.64 (14)	C12—C11—H11	120.3
C2—C1—C8	105.94 (13)	O2—C12—C11	123.55 (17)
C3—C2—C7	120.33 (15)	O2—C12—C13	116.72 (17)
C3—C2—C1	132.91 (15)	C11—C12—C13	119.72 (18)
C7—C2—C1	106.72 (13)	C14—C13—C12	119.75 (18)
C2—C3—C4	117.73 (17)	C14—C13—H13	120.1
C2—C3—H3	121.1	C12—C13—H13	120.1
C4—C3—H3	121.1	C13—C14—C15	121.26 (18)
C5—C4—C3	121.22 (17)	C13—C14—H14	119.4
C5—C4—H4	119.4	C15—C14—H14	119.4
C3—C4—H4	119.4	C10—C15—C14	118.52 (18)
C4—C5—C6	121.48 (17)	C10—C15—H15	120.7
C4—C5—H5	119.3	C14—C15—H15	120.7
C6—C5—H5	119.3	O2—C16—H16A	109.5
C7—C6—C5	117.13 (17)	O2—C16—H16B	109.5
C7—C6—H6	121.4	H16A—C16—H16B	109.5
C5—C6—H6	121.4	O2—C16—H16C	109.5
C6—C7—C2	122.09 (16)	H16A—C16—H16C	109.5
C6—C7—N1	128.44 (16)	H16B—C16—H16C	109.5
C2—C7—N1	109.47 (14)	C8—N1—C7	111.39 (14)
O1—C8—N1	127.11 (15)	C8—N1—H1	124.3
O1—C8—C1	126.47 (15)	C7—N1—H1	124.3
N1—C8—C1	106.42 (13)	C1—N2—N3	116.95 (13)
N4—C9—N3	114.35 (14)	N2—N3—C9	121.05 (13)
N4—C9—S1	128.21 (13)	N2—N3—H3A	119.5
N3—C9—S1	117.43 (12)	C9—N3—H3A	119.5
C15—C10—C11	121.28 (16)	C9—N4—C10	127.93 (14)
C15—C10—N4	118.06 (16)	C9—N4—H4A	116.0
C11—C10—N4	120.61 (15)	C10—N4—H4A	116.0
C10—C11—C12	119.44 (16)	C12—O2—C16	117.57 (15)
			
N2—C1—C2—C3	−4.6 (3)	C10—C11—C12—C13	0.8 (3)
C8—C1—C2—C3	179.95 (19)	O2—C12—C13—C14	−178.02 (19)
N2—C1—C2—C7	172.98 (16)	C11—C12—C13—C14	0.9 (3)
C8—C1—C2—C7	−2.51 (18)	C12—C13—C14—C15	−1.6 (3)
C7—C2—C3—C4	−0.8 (3)	C11—C10—C15—C14	1.2 (3)
C1—C2—C3—C4	176.49 (19)	N4—C10—C15—C14	178.50 (17)
C2—C3—C4—C5	0.0 (3)	C13—C14—C15—C10	0.6 (3)
C3—C4—C5—C6	0.4 (3)	O1—C8—N1—C7	−179.55 (17)
C4—C5—C6—C7	0.1 (3)	C1—C8—N1—C7	0.0 (2)
C5—C6—C7—C2	−0.9 (3)	C6—C7—N1—C8	177.47 (19)
C5—C6—C7—N1	−179.92 (19)	C2—C7—N1—C8	−1.6 (2)
C3—C2—C7—C6	1.3 (3)	C2—C1—N2—N3	−175.68 (15)
C1—C2—C7—C6	−176.60 (17)	C8—C1—N2—N3	−1.2 (2)
C3—C2—C7—N1	−179.52 (16)	C1—N2—N3—C9	177.49 (15)
C1—C2—C7—N1	2.6 (2)	N4—C9—N3—N2	3.9 (2)
N2—C1—C8—O1	5.7 (3)	S1—C9—N3—N2	−175.20 (12)
C2—C1—C8—O1	−178.91 (17)	N3—C9—N4—C10	178.85 (16)
N2—C1—C8—N1	−173.84 (17)	S1—C9—N4—C10	−2.1 (3)
C2—C1—C8—N1	1.57 (19)	C15—C10—N4—C9	142.08 (19)
C15—C10—C11—C12	−1.8 (3)	C11—C10—N4—C9	−40.6 (3)
N4—C10—C11—C12	−179.12 (16)	C11—C12—O2—C16	4.2 (3)
C10—C11—C12—O2	179.65 (17)	C13—C12—O2—C16	−176.89 (19)

### Hydrogen-bond geometry (Å, °)

**Table:**

*D*—H···*A*	*D*—H	H···*A*	*D*···*A*	*D*—H···*A*
N1—H1···O1^i^	0.86	2.04	2.875 (2)	164
N3—H3A···O1	0.86	2.06	2.7441 (19)	136
N4—H4A···N2	0.86	2.19	2.620 (2)	110
N4—H4A···S1^ii^	0.86	2.87	3.5806 (16)	141
C11—H11···S1	0.93	2.74	3.212 (2)	112
C8—O1···Cg3^iii^	1.2319 (19)	3.6366 (1)	3.7399 (17)	85.16 (10)

Symmetry codes: (i) −*x*, −*y*+1, −*z*+1; (ii) −*x*+1/2, *y*−1/2, −*z*+1/2; (iii) −*x*+1/2, *y*+1/2, −*z*+1/2.
